# Primary care nurses: effects on secondary care referrals for diabetes

**DOI:** 10.1186/1472-6963-10-230

**Published:** 2010-08-06

**Authors:** Christel E van Dijk, Robert A Verheij, Johan Hansen, Lud van der Velden, Giel Nijpels, Peter P Groenewegen, Dinny H de Bakker

**Affiliations:** 1 NIVEL, Netherlands Institute of Health Services Research, Utrecht, The Netherlands; 2 Department of Public and Occupational Health, VU University Medical Center, Amsterdam, The Netherlands; 3 Department of General Practice, VU University Medical Center, Amsterdam, The Netherlands; 4 Utrecht University, Department of Sociology, Department of Human Geography, Utrecht, The Netherlands; 5 Tilburg University, Scientific Centre for Transformation in Care and Welfare (TRANZO), Tilburg, The Netherlands

## Abstract

**Background:**

Primary care nurses play an important role in diabetes care, and were introduced in GP-practice partly to shift care from hospital to primary care. The aim of this study was to assess whether the referral rate for hospital treatment for diabetes type II (T2DM) patients has changed with the introduction of primary care nurses, and whether these changes were related to the number of diabetes-related contacts in a general practice.

**Methods:**

Healthcare utilisation was assessed for a period of 365 days for 301 newly diagnosed and 2124 known T2DM patients in 2004 and 450 and 3226 patients in 2006 from general practices that participated in the Netherlands Information Network of General Practice (LINH). Multilevel logistic and linear regression analyses were used to analyse the effect of the introduction of primary care nurses on referrals to internists, ophthalmologists and cardiologists and diabetes-related contact rate. Separate analyses were conducted for newly diagnosed and known T2DM patients.

**Results:**

Referrals to internists for newly diagnosed T2DM patients decreased between 2004 and 2006 (OR:0.44; 95%CI:0.22-0.87) in all practices. For known T2DM patients no overall decrease in referrals to internists was found, but practices with a primary care nurse had a lower trend (OR:0.59). The number of diabetes-related contacts did not differ between practices with and without primary care nurses. Cardiologists' and ophthalmologists' referral rate did not change.

**Conclusions:**

The introduction of primary care nurses seems to have led to a shift of care from internists to primary care for known diabetes patients, while the diabetes-related contact rate seem to have remained unchanged.

## Background

Primary care nurses have established their position in general practice in several countries in the last decades. They play an increasingly important role in the care of type 2 diabetes (T2DM) patients, and in health promotion and routine management of these patients[1-3]. In the United Kingdom, Sweden and Finland, where nurses have traditionally been involved in primary care, their roles have widened in the last decade[4-6], and in countries with no tradition of nurses in general practice, like the Netherlands and Australia, nurses have been gradually introduced[7,8].

The introduction or extension of the tasks of primary care nurses can be stimulated by the introduction of new contracts and regulations by governments. Three examples of countries where new contracts and regulation for primary care nurses were implemented are the United Kingdom, Australia and the Netherlands. In the United Kingdom, the introduction in 2004 of the Quality and Outcomes Framework (QOF) within the New General Medical Services Contract has resulted in an extension of the activities of primary care nurses in the management of chronic illnesses such as asthma and diabetes[5,9].

In Australia, the introduction of the Practice Incentives Program (PIP) with the Practice Nurse Incentive (PNI) in 2001 has encouraged general practices in rural and remote areas to employ primary care nurses. Primary care nurses must be engaged in a variety of activities including patient education, acute and chronic disease management, diagnostic services and clinical data management[10,11].

In the Netherlands, primary care nurses were introduced in the late nineties and were predominantly involved in care for chronically ill patients. Initially the increase in the number of primary care nurses was gradually and stopped when health insurance companies stopped providing new contracts for primary care nurses in 2004 (Additional file 1). In 2006 new contracts were provided again and the funding system altered. Care provided by primary care nurses is funded from consultation fees equal to those of GPs whereas before 2006 primary care nurses were only funded from with a small supplement on the capitation fee for publicly insured patients (67% of the population). These measures have probably been the driving force behind the growth in primary care nurses in general practice between 2003 and 2007.

Reasons for stimulating the role of nurses can be found in the increasing demand for primary care services, combined with concerns about the supply of physicians and the increased pressure to contain costs[2,12]. Higher demand for primary care services is the result of an ageing population, rising patient expectations, a growing number of chronically ill patients and the desire to shift care from hospital to primary care[12,13]. Literature suggests that in general primary care nurses provide the same quality of care as general practitioners (GPs)[12-17], but have not resulted in a lower workload for GPs[18]. Primary care nurses seem to have strengthened primary care, especially for chronically ill patients. However, primary care costs have increased. These extra costs may be justified if the introduction of primary care nurses would result in a shift of care from hospital care to general practice, i.e. substitution, or has improved the quality of care. The Dutch situation provides a good test case for this hypothesis for diabetes type 2 (T2DM) patients since primary care nurses have been providing care to T2DM patients since the introduction.

Sibbald et al. (2004) stated in their review that changing workforce skill-mix is one strategy to improve the effectiveness and efficiency of healthcare[17]. Changes in skill-mix may be brought about through enhancement, substitution, delegation or innovation. Research on substitution in general is, however, restricted to effects within primary care, and no previous research has addressed the possible effects of primary care nurses in terms of shifting care from hospital care to general practice [19].

Since GPs act as gatekeepers in the Dutch healthcare system, referral rates can be used to measure substitution. The first research question to be answered is:

Did the referral rate for hospital treatment change for T2DM patients between 2004 and 2006 with the introduction of primary care nurses in general practice?

The years 2004 and 2006 were chosen since the increase in the number of primary care nurses working in general practice occurred in this timeframe, which enables us to compare practices with and without primary care nurses. The effect of the introduction of primary care nurses was expected to be different for internists, cardiologists, ophthalmologists, and mental healthcare. We expected the referrals to internists and cardiologists to be reduced with the introduction of primary care nurses, since primary care nurses generally follow the guidelines and generally provide more repeat consultations, which may result in better quality of care[12,20]. Possible complications and comorbid conditions would be detected earlier and would be managed more often within general practice. Mental healthcare was included, since T2DM patients appeared to have a higher change of depression[21]. For referrals to mental healthcare, our hypothesis was that patients within a practice with primary care nurses would be less often referred to mental healthcare since primary care nurses' consultation time is generally longer than that of GPs[12], which could influence time involved in social support in the management of T2DM. Regarding the use of care by ophthalmologists an opposite effect was expected, since guidelines recommend yearly referral of T2DM patients to ophthalmologists for eye fundus examination if expertise to examine the eye fundus is not available in general practice[1].

Differences in trend found in referral rates could be an indirect effect of a higher consultation rate for diabetes within practices with primary care nurses. More consultations may lead to a better regulation of diabetes and quality of care. For that reason, we additionally answered the following research question:

Is the diabetes-related contact rate higher in practices with primary care nurses?

## Methods

### Study design

In the Netherlands GPs are supposed to treat patients themselves unless referral to a medical specialist or other healthcare provider is needed. About 90% of all health problems presented in GP-practice is treated by the GP self. Referral rates to medical specialists thus are an indicator for what can be handled in general practice and what not. GPs provide community based family medicine and internists hospital based internal medicine.

To answer the research questions we analysed whether the referral rate to internists, ophthalmologists, cardiologists and mental healthcare changed from 2004 to 2006, and whether or not this was different for general practices with and without primary care nurses. We also examined whether the diabetes-related contact rate was different in practices with and without primary care nurses. The diabetes-related contact rate was only analysed in 2006, since no detailed information was available for 2004. A distinction was made between newly diagnosed and known T2DM patients. To convert the treatment of patients to primary care nurses is harder for patients who have been treated by GPs or internists for years, than for newly diagnosed patients.

For the purpose of this study, we used data on healthcare utilization of newly diagnosed and known T2DM patients for a period of 365 days after the first diagnosis of T2DM (newly diagnosed T2DM patients) or after the first consultation or prescription for T2DM (known T2DM patients) in 2004 and 2006. T2DM patients were seen as newly diagnosed when patients had no diabetes record in GPs' electronic medical record (EMR) in the previous years (with minimum of one year). In total, 450 newly diagnosed and 3226 known T2DM patients in 2006 and 301 newly diagnosed and 2124 known T2DM patients in 2004 were included in the analyses.

### Subjects

Data were derived from EMRs of general practices that participated in the Netherlands Information Network of General Practice (LINH)[22]. LINH is a representative sample of general practices in the Netherlands. Each year some minor changes in composition of practices occur due to natural turnover. The data hold information about morbidity (international classification of primary care (ICPC codes)[23]), prescriptions, contacts and referrals. Medical ethical approval was not required for this research.

**Figure 1:**
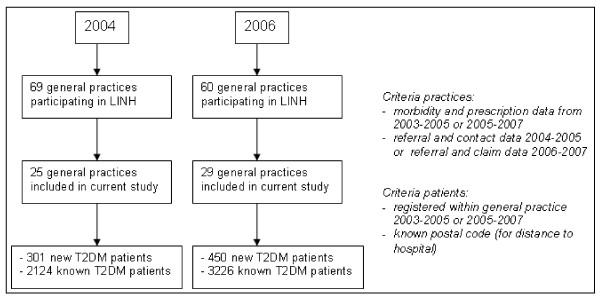
**Flow chart of general practices and patients included in the study**.

Figure 1 shows the inclusion criteria for general practices and patients in 2004 and 2006 and the number of practices and patients included. In 2004 25 practices and in 2006 29 practices were included. Most practices were excluded from the analyses owing to a poor recording of referrals. The selection of practices forms a representative sample of Dutch general practices with regard to practice type (single handed, duo, group or health centre), degree of urbanisation and province.

T2DM patients were selected on the basis of the ICPC code for diabetes: T90. We were not able to distinguish T2 and T1 diabetes patients on the basis of the ICPC-coding. For the purpose of this study, type I diabetes patients were excluded on the basis of prescription data (ATC-coded[24]). Type I diabetes was characterised by diabetes patients with a prescription of insulin (ATC code A10A), but without oral anti-diabetic medication (ATC code A10B)[25].

### Measurements

#### Referrals

We analysed new referrals to internists, ophthalmologists, cardiologists or mental healthcare. A patient was considered as being referred (1) if a referral had been recorded within 365 days after the first diagnosis or first consultation for diabetes (including this consultation). Referrals to mental healthcare included referrals to psychiatrists, psychologists or ambulatory mental healthcare.

It was unknown whether GPs could perform an eye fundus examination in their own practice and therefore not refer patients to ophthalmologists. Most of these GPs, however, probably perform only retinaphotography and leave the examination of this photo to ophthalmologists.

#### Diabetes-related contacts with general practice

Diabetes-related contacts were only assessed in 2006 and based on the number of claimed telephone and office consultations and home visits with an ICPC code T90 (diabetes). In 85.8% of all consultations and home visits the diagnosis was known in 2006 and 2007.

#### Primary care nurses

The presence of a primary care nurse was determined for all general practices on the basis of data from the EMR.

##### Covariates

We adjusted for factors that could affect the relation between referral rate and presence of a primary care nurse or the relation between diabetes contact and presence of a primary care nurse. These included comorbidity and distance to hospital apart from gender and age (continuous).

#### Comorbidity

Comorbidity was taken as covariate, since T2DM patients with comorbidity were assumed to be more likely to be referred to a medical specialist than patients without comorbidity[26] and may have more consultations. Using the ICPC codes in the EMR of the practices, we distinguished between diabetes-related comorbidity and unrelated comorbidity. Related comorbidity included heart diseases, stroke, retinopathy, nephropathy and diabetic foot. Non-related comorbidity included depression, lung diseases, musculoskeletal diseases, neurological diseases and cancer. Additional file 2 shows the ICPC codes and descriptions. Patient were regarded having related or unrelated comorbidity (0/1) if s/he had consulted the GP or had a prescription for one of these diseases.

#### Distance to hospital

Distance to the nearest hospital for a patient might influence the referral behaviour of GPs, since they might be more reluctant to refer patients living further away from a hospital[27]. Road distance to the nearest hospital was based on distance from the centroid of the postal code of the patient's home to the nearest hospital.

### Statistical analyses

To analyse the relation of the presence of primary care nurses with contacts with general practice and change in referral in T2DM patients, multilevel logistic regression analyses (referrals) and multilevel linear regression analyses (contacts) were conducted with MLwiN 2.02. Multilevel analysis corrects for the cluster effect of patients within general practices[28].

In analyses of referral rates between 2004 and 2006, time was included as a dummy variable representing 2006, with 2004 as reference category. For all analyses, first a model with only the dependent variables was analysed (model 1). Second, covariates were added to the model (model 2). Last, the interaction term 'primary care nurse in practice*year' was added to the referral analyses (model 3). Covariates in the referral analyses were age, gender, and related and unrelated comorbidity and distance to hospital. Covariates in the contact analyses were age, gender, and related and unrelated comorbidity. In addition, the effect of primary care nurses in practice was analysed separately for 2004 and 2006.

Analyses of referrals were performed separately for referrals to internists, ophthalmologists, cardiologists and mental healthcare. The significance level was set at p < 0.05. For the interaction 'primary care nurse in practice* year', significance level was set at p < 0.10 since this was measured on practice level and the number of practices is much smaller than the number of patients. The models were estimated with multilevel logistic regression analyses with second-order PQL (penalised quasi-likelihood), and multilevel linear regression analyses, both with only a random intercept.

## Results

### Patient characteristics

In 2006, 39.6 per 1000 patients in general practice were identified with T2DM, 4.9 per 1000 of whom were diagnosed for the first time. For 2004, this was 33.1 and 4.1 per 1000 patients respectively. 72% (N = 21) of the general practices in 2006 and 52% (N = 13) of the practices in 2004 had a primary care nurse. Table 1 shows the patient characteristics of the newly diagnosed and known T2DM patients in 2006.

**Table 1:** Patient characteristics and healthcare in 2006 and uncorrected number of referrals to internists, ophthalmologists, cardiologists and mental healthcare in 2004 and 2006 for newly diagnosed and known diabetes patients.

	Newly diagnosed diabetes patients	Known diabetes patients
**Patient characteristics**		
Gender^2 ^(male)	50.2% (226)	47.2% (1705)
Age^1 ^(in years)	61.4 (SD:14.1)	67.1 (SD:11.9)
Distance to hospital^1 ^(km)	8.6 (SD:6.9)	8.3 (SD:7.1)
Related comorbidity^2^	19.1% (86)	19.6% (633)
Unrelated comorbidity^2^	39.3% (177)	35.1% (1133)
		
**Healthcare utilisation in 2006**		
Diabetes guidance per year^2^	21.1% (95)	17.8% (575)
Number of diabetes contacts		
Total^1^	1.8 (SD:1.04)	1.8 (SD: 0.98)
PCN-practice^1^	1.8 (SD:1.05)	1.8 (SD: 0.99)
Non-PCN-practice^1^	1.7 (SD:1.02)	1.6 (SD: 0.90)
		
**Referral rates**		
Internist^2^		
2004	7.3% (22)	5.7% (121)
2006	3.3% (15)	4.9% (158)
Ophthalmologist^2^		
2004	25.2% (76)	10.4% (221)
2006	29.1% (131)	12.8% (413)
Cardiologist^2^		
2004	2.3% (7)	3.1% (66)
2006	3.3% (15)	3.1% (98)
Mental healthcare ^2^		
2004	0.7% (2)	1.0% (22)
2006	1.8% (8)	0.7% (24)

^1^mean (standard deviation); ^2 ^percentage (number)

In 2006, 19.1% of the newly diagnosed T2DM patients and 19.6% of the known T2DM patients had related comorbidity and 39.3% and 35.1% respectively had unrelated comorbidity. The commonest diabetes-related comorbidity was heart disease (14.4% and 15.3%). For unrelated comorbidity, musculoskeletal diseases (28.4% and 25.1%) were the commonest, followed by lung diseases (10.0% and 8.7%). Patient characteristics did not differ significantly between 2004 and 2006, with the exception of related comorbidity in newly diagnosed T2DM patients. The number of patients with related comorbidity was higher in 2006 (19.1%) than in 2004 (13.6%).

### Changes in referral rates between 2004 and 2006

Table 1 also presents the referral rates to internists, ophthalmologists, cardiologists and mental healthcare for newly diagnosed and known T2DM patients in 2004 and 2006. Referral rates were low for internists (5.3% for newly diagnosed and known T2DM patients) and seem to have decreased for newly diagnosed T2DM patients between 2004 and 2006. Newly diagnosed patients, not surprisingly, were referred twice as often to ophthalmologists as known T2DM patients (on average 27.2% vs. 11.6%). Furthermore, T2DM patients were hardly ever referred to mental health services: on average 1.3% for newly diagnosed patients and 0.9% for known T2DM patients. Due to the very low referral rate to mental health services, no further analyses were performed for mental healthcare.

Table 2 shows the results from multilevel logistic regression analyses for referrals to internists, ophthalmologists and cardiologists between 2004 and 2006. The referral rate for newly diagnosed T2DM patients to internists decreased more than 50% between 2004 and 2006 (OR:0.44; 95%CI:0.22-0.88;P < 0.05). However, the trend in referral rate between 2004 and 2006 did not differ between general practices with and without primary care nurses. The referral rate to internists for known T2DM patients did not change between 2004 and 2006 (OR:0.85;95%CI:0.65-1.11 P = 0.23). However, in general practices with a primary care nurse the trend in referrals to internists was lower than in general practices without a primary care nurse (OR:0.59;P < 0.1)

**Table 2:** Multilevel logistic regression analyses with dependent variables referrals to internists, ophthalmologists and cardiologists and as independent variable time and presence of primary care nurse for newly diagnosed and known diabetes patients in 2004 to 2006^§^.

	Newly diagnosed diabetes patients						Known diabetes patients					
	
	Model 1		**Model 2**^**#**^		**Model 3**^**# ‡**^		Model 1		**Model 2**^**#**^		**Model 3**^**# ‡**^	
	
	OR	95% CI	OR	95% CI	OR	95% CI	OR	95% CI	OR	95% CI	OR	95% CI
Internist												
Difference 2004-2006	**0.44**	**0.22-0.88^1^**	**0.42**	**0.21-0.85^1^**	**0.12**	**0.02-1.00^1^**	0.85	0.65-1.10	0.87	0.66-1.14	1.31	0.76-2.28
Primary care nurse			1.24	0.58-2.67	0.87	0.35-2.12			0.85	0.60-1.21	1.11	0.71-1.75
PCN*Year*					4.52	0.48-42.32					**0.59**	**0.31-1.11^2^**

Ophthalmologist												
Difference 2004-2006	1.14	0.70-1.87	1.21	0.71-2.07	1.65	0.49-5.59	0.93	0.73-1.17	0.98	0.76-1.25	1.41	0.71-2.81
Primary care nurse			0.79	0.36-1.74	0.93	0.35-2.43			0.79	0.49-1.27	0.91	0.53-1.55
PCN*Year*					0.67	0.17-2.74					0.65	0.31-1.38

Cardiologist												
Difference 2004-2006	1.45	0.58-3.59	1.99	0.74-5.31	2.59	0.67-10.04	1.00	0.70-1.41	1.00	0.70-1.42	1.08	0.50-2.34
Primary care nurse			**0.30**	**0.12-0.78^1^**	0.43	0.09-1.99			0.87	0.55-1.38	0.91	0.49-1.71
PCN*Year^*^					0.58	0.09-3.88					0.91	0.37-2.19

^§^For both newly diagnosed and known diabetes patient separate analyses were performed for referrals to internists, ophthalmologists and cardiologist. This table shows the results of 18 analyses.

^#^adjusted for age, gender, distance to hospital, related and unrelated comorbidity

^‡^in addition primary care nurse included as variable in the analysis

*interaction term presence primary care nurse and difference 2004-2006

^1^significant p < 0.05

^2^significant p < 0.1

The trend in referral rate to ophthalmologists and cardiologists for newly diagnosed and known T2DM patients did not show any difference between 2004 and 2006 nor were differences found between general practices with and without a primary care nurse.

The presence of primary care nurses was not related to the referral rate to internists and ophthalmologists in both 2004 and 2006 together, but did affect the referral rate to cardiologists in newly diagnosed diabetes patients (OR:0.30; 95%CI:0.12-0.78; P < 0.05). Surprisingly, the effect of primary care nurses in general practice changed between 2004 and 2006 (Table 3). The presence of a primary care nurse affected the referral rate to internists for known T2DM patients in 2006 (OR: 0.61; 95% CI:0.39-0.95;P < 0.05), but not in 2004 (OR: 1.25; 95% CI: 0.80-1.96; P = 0.33). The presence of primary care nurses in general practice did not affect the referral rate to ophthalmologists and cardiologists in either 2004 or 2006.

**Table 3:** Multilevel logistic regression analyses with dependent variables referrals to internist, ophthalmologist and cardiologist and as independent variable presence of primary care nurse for newly diagnosed and known diabetes patients, 2004 and 2006^§^.

	Newly diagnosed diabetes patients				Known diabetes patients			
	
	Model 1		**Model 2**^**#**^		Model 1		**Model 2**^**#**^	
	
	OR	95% CI	OR	95% CI	OR	95% CI	OR	95% CI
*2004*								
Internist	0.75	0.25-2.21	0.77	0.27-2.13	1.22	0.78-1.91	1.25	0.80-1.96
Ophthalmologist	2.09	0.59-7.35	1.84	0.56-6.08	1.36	0.67-2.72	1.29	0.65-2.58
Cardiologist	0.45	0.10-2.04	0.45	0.09-2.20	1.07	0.50-2.31	0.97	0.49-1.94
*2006*								
Internist	3.65	0.47-28.09	4.23	0.53-34.07	0.70	0.45-1.08	**0.61**	**0.39-0.95^1^**
Ophthalmologist	0.85	0.19-3.87	1.85	0.39-8.66	0.62	0.24-1.59	0.69	0.27-1.74
Cardiologist	0.36	0.13-1.05	0.30	0.09-1.00	0.91	0.46-1.79	0.68	0.36-1.30

^§^For both newly diagnosed and known diabetes patient separate analyses were performed for referrals to internists, ophthalmologists and cardiologist and for both 2004 and 2006 This table shows the results of 24 analyses.

^1^significant P < 0.05

^#^referral analyses adjusted for age, gender, distance to hospital, related and unrelated comorbidity;

### Contact with general practice

Table 4 presents the results of the multilevel linear regression analyses for diabetes-related contacts with general practices. The presence of a primary care nurse in a general practice was not related to the number of diabetes-related contacts in either known or newly diagnosed T2DM patients.

**Table 4:** Multilevel linear regression analyses with dependent variable diabetes-related contacts with the general practice and as independent variable presence of primary care nurse for newly diagnosed and known diabetes patients, 2006^§^.

	Newly diagnosed diabetes patients				Known diabetes patients			
	
	Model 1		**Model 2**^**#**^		Model 1		**Model 2**^**#**^	
	
	β	95% CI	β	95% CI	β	95% CI	β	95% CI
Primary care nurse	0.04	-0.35-0.43	0.04	-0.35-0.43	0.04	-0.31-0.38	0.03	-0.32-0.38

^§^For both newly diagnosed and known diabetes patient the number of diabetes-related contact with general practice was compared for practices with and without a primary care nurse.

^#^analyses adjusted for age, gender, related and unrelated comorbidity

## Discussion

The aim of this study was to assess whether the referral rate for hospital treatment changed for T2DM patients with the introduction of primary care nurses in general practice and whether such effects could be due to an increase in contact rate for diabetes in general practices with a primary care nurse.

On average, referral rates of newly diagnosed and known diabetes patients to internists (both 5.3%), cardiologists (2.8% and 3.1%) and mental healthcare (1.3% and 0.9%) were low. Referrals to ophthalmologists were more common and higher for newly diagnosed diabetes patients (27.2% vs. 11.6%). The referral rate to internists for newly diagnosed T2DM patients decreased in general practices both with and without a primary care nurse between 2004 and 2006, and the trend in referral rate to internists between 2004 and 2006 for known T2DM patients was lower in general practices with primary care nurses than in general practices without primary care nurses. The difference in trend in referrals to internists for known T2DM patients did not seem to be related to a higher contact rate for diabetes in general practices with primary care nurses, since the diabetes-related contact rate did not differ between practices with and without a primary care nurse. The referrals to ophthalmologists and cardiologists for both newly diagnosed and known diabetes patients did not change between 2004 and 2006.

### Strengths and limitations of the study

LINH provides a dataset based on consultations in general practice with data on diagnosis, treatment and referrals, as a result of which we could measure the effect of primary care nurses on referral rate in T2DM patients. This study had some limitations. General practices were selected on the basis of the quality of their EMR, and therefore selection bias may have occurred. The selection bias affected the registration of data in GPs' EMR as such, but was not expected to affect referral rates. Our study was an observational study, with no randomisation of general practices with and without primary care nurses. The results of our study could, therefore, be caused by other factors influencing the employment of primary care nurses. Primary care nurses were first introduced before our research timeframe. We are of the opinion that the period between 2004 and 2006 provides a good insight into the effect of primary care nurses, since the number of whole time equivalents primary care nurses working in general practices increased tremendously between 2004 and 2006. Primary care nurses have been providing care to T2DM patients since the introduction, and therefore much experience has been gained. Further, the number of contacts was based on claims data, so contacts which were not claimed were not taken into account. In the Netherlands, general practices are reimbursed € 9-18 for consultations and €13.5-22.5 for home visits. Therefore, their income is dependent on recording of contacts. For this reason it is not likely that many contacts have been missed. For known diabetes patients referrals outside the one-year period were not available and could have influenced the results. For newly diagnosed T2DM patients, this is no problem since patients need a referral to be treated by internists. Moreover, we had no other indications in the records of the GPs that the T2DM patients were under treatment by internists. Finally, we have analysed the effect of the presence of primary care nurses on referral to a specialist. This gives no indication about the number of consultations with medical specialists. For instance, it could be that T2DM patients in 2006 were only referred for one consultation with a medical specialist.

### Comparison with other research

Our results suggest that the introduction of primary care nurses in general practice may have resulted in a shift from care by internists to care by general practice for known diabetes patients. The shift of care from internists to general practice could not be linked to a higher number of diabetes-related contacts in general practices with a primary care nurse. The decrease in referral rate can also not be explained by an overall decrease in referral rate to internists, since the overall referral rate to internists was stable between 2004 and 2006[22]. The number of diabetes-related contacts was not higher in practices with or without a primary care nurse. This seems to be contrary to results in the literature about contact rates of primary care nurses[12]. In our study, the diabetes-related contact rate in practices with primary care nurses consisted of contacts of T2DM patients treated by primary care nurses or GP, whereas other studies analysed diabetes-related contact rate of patients treated only by primary care nurses. Analyses within general practices with primary care nurses showed, however, higher consultation rates for diabetes patients under treatment by primary care nurses, which is in accordance with the literature (not shown). Still the average consultation rate of diabetes patients is low in our population in comparison to the diabetes guidelines which advices at least 4 contacts per year for diabetes[1]. Therefore, other characteristics of the care provided by practices with primary care nurses must have brought about this trend in referrals to internists. A recent study showed that diabetes patients more often receive optimal care, in terms of diabetes-related examinations, in primary care when a diabetes education programme is available or when yearly medical check-ups are done by both the GP and primary care nurse[29]. Further, primary care nurses generally provide longer consultations[12], which may positively affects the quality of care for T2DM patients. Another intriguing finding for the referrals to internists was that the presence of primary care nurses affected the referrals to internists in 2006, but not in 2004. This was probably due to the increased number of primary care nurses working in general practices and their experience with diabetes care.

Our study showed no difference in referrals to cardiologists after the introduction of primary care nurses in general practice, whereas we expected the referral rate to be lower in practices with primary care nurses. The reason could lie in the low overall referral rate of T2DM patients to cardiologists. The referral rate to cardiologists was lower in practices with primary care nurses in 2004 and 2006 in newly diagnosed patients, but the difference in referral between 2004 and 2006 was for practices with and without primary care nurses non-significant. A recent study found indications that primary care nurses follow the guidelines better for cardiovascular risk management than GPs. This study found a decrease in the mean level of risk factors in high-risk patients after 1 year of cardiovascular management, with a larger decrease in patients allocated to primary care nurses[20].

For ophthalmologists, we expected the referral rate to have increased after the introduction of primary care nurses, since guidelines recommend yearly referral to ophthalmologists for eye fundus examination, if expertise to examine the eye fundus is not available in general practice. Our results, however, showed no differences in the referral rate to ophthalmologists. Our results could be biased because some general practices may perform an eye fundus examination themselves or patients visit the ophthalmologist without a referral. Results from the Panel of Patients with Chronic Diseases (NPCD), based on patients' recall, showed that in the Netherlands 42% of the T2DM patients in 1998 visited an ophthalmologist[30] and 63% of T2DM patients visited one in 2008. In this study, we found a referral rate of 15% of the total T2DM population.

This study also found a higher prevalence of diabetes in 2006 compared to 2004. This higher prevalence might be explained by the increased attention for screening in general practice in 2006. In the Netherlands several campaign have taken place to stimulate the screening for diabetes in 2006.

## Conclusions

Our results suggest that the introduction of primary care nurses may have resulted in a shift in care from internists to primary care for known diabetes patients. This did not seem to be explained by more contacts for diabetes in practices with primary care nurses.

## Competing interests

The authors declare that they have no competing interests.

## Authors' contributions

CD, RV, LV, GN, PG, DB were involved in the conception of the research question. CD and JH were involved in analysing the data. All authors had full access to all the data and contributed to the interpretation of the data. CD drafted the manuscript, which was reviewed by all authors.

## Pre-publication history

The pre-publication history for this paper can be accessed here:

http://www.biomedcentral.com/1472-6963/10/230/prepub

## Supplementary Material

Additional file 1 **Introduction of primary care nurses in the Netherlands**. Additional file 1 provides a description of the introduction of primary care nurses in the Netherlands. Click here for file

Additional file 2 **ICPC description codes related and unrelated comorbidity**. Additional file 2 provides a full description of the ICPC-codes used for the determination of related and unrelated comorbidity. Click here for file
