# Supplementary material for: Primary care nurses: effects on secondary care referrals for diabetes
Source: BMC Health Serv Res. 2010 Aug 6;10:230. doi: 10.1186/1472-6963-10-230 (PMC2924333; doi:10.1186/1472-6963-10-230)
Supplement: Additional file 1 — Introduction of primary care nurses in the Netherlands. Additional file 1 provides a description of the introduction of primary care nurses in the Netherlands. [file 1472-6963-10-230-S1.DOC]

**Additional file 1: Introduction of primary care nurses in the Netherlands**

| Primary care nurses were introduced in 1999 and need to have a bachelor degree in nursing or are practice assistances who have followed extra education. They are predominantly involved in the care of chronically ill patients. In 2001, funding for GP practices to employ primary care nurses was introduced. General practices with a primary care nurse received a supplement on the capitation fee for publicly insured patients (67%) and could claim a consultation fee for primary care nurses for privately insured patients. The introduction of primary care nurses went slowly with in 2002 400 WTE primary care nurses working for 25% of GPs. In 2004, the gradual implementation stopped. Public health insurers did not provide new contracts for primary care nurses, but existing contracts were not cancelled. In 2006, a single universal basic health insurance was implemented for the whole population, abolishing the difference between privately and publicly insured patients. With the revised health insurance system, funding of primary care nurses changed to a mixed system for all patients with a supplement on the capitation fee and a fee-for-service. General practices could again arrange new contracts with health insurers for the employment of primary care nurses. In 2007, 60% of GPs had a primary care nurse working in the practice, an increase of 60% from 2003. More remarkably, the number of WTE primary care nurses working in a general practice increased with 149% between 2003 and 2007 from 1,100 WTE to 2,700 WTE.  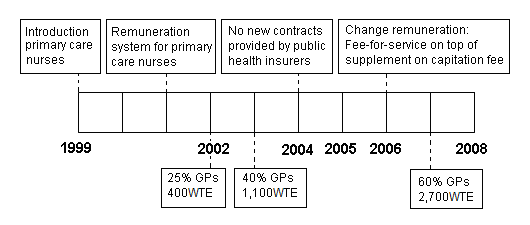 |
| --- |
