# Supplementary material for: Primary care nurses: effects on secondary care referrals for diabetes
Source: BMC Health Serv Res. 2010 Aug 6;10:230. doi: 10.1186/1472-6963-10-230 (PMC2924333; doi:10.1186/1472-6963-10-230)
Supplement: Additional file 2 — ICPC description codes related and unrelated comorbidity. Additional file 2 provides a full description of the ICPC-codes used for the determination of related and unrelated comorbidity. [file 1472-6963-10-230-S2.DOC]

Additional file 2: ICPC description codes related and unrelated comorbidity

| **ICPC-code** | **Description** |
| --- | --- |
| *Related comorbidity* |  |
| F83 | Retinopathy |
| K74 | Angina pectoris |
| K75 | Acute myocardial infarction |
| K76 | Ischaemic heart disease |
| K90 | Stroke/ Cerebrovascular accident (CVA) |
| S97 | Chronic ulcer skin |
| U99 | Urinary disease other |
| *Unrelated comorbidity* |  |
| B74 | Malignant neoplasm blood other |
| D74 | Malignant neoplasm stomach |
| D75 | Malignant neoplasm colon/rectum |
| D77 | Malignant neoplasm digest other/NOS |
| L01 | Neck symptom/complain |
| L02 | Back symptom/complaint |
| L03 | Low back symptom/complaint |
| L08 | Shoulder symptom/complaint |
| L13 | Hip symptom/complaint |
| L15 | Knee symptom/complaint |
| L84 | Osteoarthritis spine |
| L86 | Back syndrome with radiating pain |
| L89 | Osteoarthrosis of hip |
| L90 | Osteoarthrosis of knee |
| L91 | Osteoarthrosis other |
| N86 | Multiple sclerosis |
| N87 | Parkinsonism |
| N88 | Epilepsy |
| P76 | Depressive disorder |
| R84 | Malignant neoplasm bronchus/lung |
| R91 | Chronic bronchitis/bronchiectasis |
| R95 | Chronic obstructive pulmonary disease |
| R96 | Asthma |
| S77 | Malignant neoplasm of skin |
| X76 | Malignant neoplasm breast female |
| Y77 | Malignant neoplasm prostate |
